# Long Noncoding RNA JHDM1D-AS1 Promotes Tumor Growth by Regulating Angiogenesis in Response to Nutrient Starvation

**DOI:** 10.1128/MCB.00125-17

**Published:** 2017-08-28

**Authors:** Ayano Kondo, Aya Nonaka, Teppei Shimamura, Shogo Yamamoto, Tetsuo Yoshida, Tatsuhiko Kodama, Hiroyuki Aburatani, Tsuyoshi Osawa

**Affiliations:** a Division of Genome Science, RCAST, The University of Tokyo, Tokyo, Japan; b Innovative Technology Laboratories, Kyowa Hakko Kirin Co., Ltd., Tokyo, Japan; c Department of Systems Biology, Graduate School of Medicine, Nagoya University, Nagoya, Japan; d Translational Research Unit, Kyowa Hakko Kirin Co., Ltd., Shizuoka, Japan; e Laboratory for Systems Biology and Medicine, RCAST, The University of Tokyo, Tokyo, Japan

**Keywords:** angiogenesis, cancer, epigenetics, long noncoding RNA, nutrient starvation, tumor microenvironment

## Abstract

Long noncoding RNAs play a pivotal role in tumor progression, but their role in cancer cells in the nutrient-starved tumor microenvironment remains unknown. Here, we show that a nutrient starvation-responsive long noncoding RNA, JHDM1D antisense 1 (JHDM1D-AS1), promotes tumorigenesis by regulating angiogenesis in response to nutrient starvation. Expression of JHDM1D-AS1 was increased in cancer cells. In addition, expression of JHDM1D-AS1 was increased in clinical tumor samples compared to that in normal tissue. Stable expression of JHDM1D-AS1 in human pancreatic cancer (PANC-1 and AsPC-1) cells promoted cell growth *in vitro*. Remarkably, these JHDM1D-AS1-expressing cells showed a significant increase in tumor growth *in vivo* that was associated with increased formation of CD31^+^ blood vessels and elevated infiltration of CD11b^+^ macrophage lineage cells into tumor tissues. Genome-wide analysis of tumor xenografts revealed that expression of genes for tumor-derived angiogenic factors such as h*HGF* and h*FGF1* concomitant with host-derived inflammation-responsive genes such as m*Mmp3*, m*Mmp9*, m*S100a8*, and m*S100a9* was increased in tumor xenografts of JHDM1D-AS1-expressing pancreatic cancer cells, leading to a poor prognosis. Our results provide evidence that increased JHDM1D-AS1 expression under nutrient starvation accelerates tumor growth by upregulating angiogenesis, thus laying the foundation for improved therapeutic strategies.

## INTRODUCTION

The advent of next-generation sequencing and progress in transcriptome analysis has revealed that a majority of transcripts are not protein-coding RNAs but rather noncoding RNAs (ncRNAs) (1). Among ncRNAs, long ncRNAs (lncRNAs) that are >200 bases long have been reported to interact with DNA-binding proteins, such as chromatin-modifying complexes and transcription factors, and regulate gene expression through epigenetic alterations in the nucleus (2, 3) or to function as a molecular sponge in the cytoplasm (4). Recent studies have shown that lncRNAs play important roles in various aspects of tumor progression, such as metastasis and angiogenesis, partly under hypoxia (2, 5).

Angiogenesis plays an essential role in tumor growth and metastasis (6, 7). Cross talk between cancer cells and immune cells via cytokines enhances macrophage infiltration and inflammatory responses, promoting angiogenesis (8). Key angiogenic stimuli are vascular endothelial growth factors (VEGFs), fibroblast growth factors (FGFs), and hepatocyte growth factor (HGF) (9, 10).

We previously reported that histone demethylase Jumonji C domain containing histone demethylase 1 homolog D (JHDM1D, also known as KDM7A) was highly induced in cancer cells under nutrient starvation and decreased tumor growth by regulating tumor angiogenesis (11); however, the specific lncRNAs potentially involved in angiogenesis under nutrient starvation stress are largely unknown.

In this study, we identified the nutrient starvation-responsive lncRNA JHDM1D antisense 1 (JHDM1D-AS1), which arises from the antisense strand of JHDM1D. We demonstrated the role of JHDM1D-AS1 in tumor progression using cancer cells stably expressing JHDM1D-AS1 and found that JHDM1D-AS1 increases tumor growth by regulating tumor angiogenesis and may lead to a poor prognosis.

## RESULTS

### JHDM1D-AS1 is highly expressed in cancer cells and tumor tissues under nutrient starvation.

**FIG 1:**
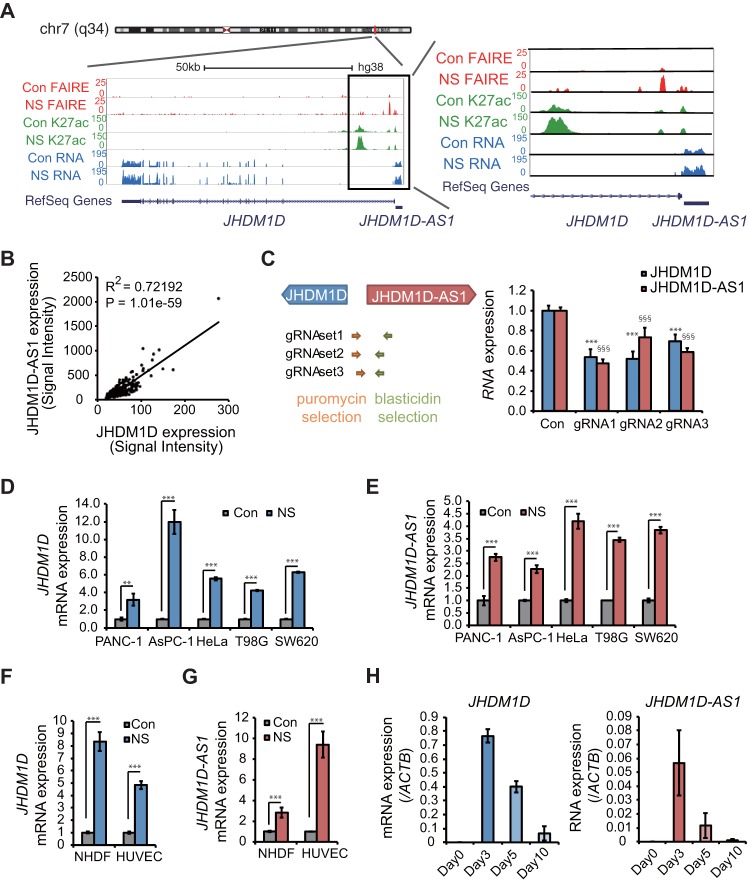
JHDM1D-AS1 is coexpressed with JHDM1D under nutrient starvation. (A) JHDM1D-AS1 and JHDM1D share a promoter at chr 7. The histone H3K27ac marks and open chromatin region comprise the shared promoter. FAIRE-seq, H3K27Ac ChIP-seq, and RNA seq were conducted in PANC-1 cells under nutrient starvation (NS) in comparison to the nutrient-rich control (CON) conditions. (B) JHDM1D-AS1 RNA expression levels are highly correlated with JHDM1D levels in various cancer cell lines (the expression data were obtained from Affymetrix Exon array data obtained from our institutional database, RefExA [http://www.lsbm.org/site_e/database/index.html]). Pearson's correlation test was used (*P* < 0.05 for significance; *r* = correlation coefficient). (C) CRISPR/Cas-mediated genomic deletion of the JHDM1D-AS1 promoter region downregulates the expression of both JHDM1D-AS1 and JHDM1D. A schematic of the genomic target regions is shown on the left. (D) The expression level of JHDM1D is increased in response to nutrient starvation in PANC-1, AsPC-1, HeLa, T98G, and SW620 cells. (E) The expression level of JHDM1D-AS1 is increased in response to nutrient starvation in PANC-1, AsPC-1, HeLa, T98G, and SW620 cells. (F) The expression level of JHDM1D is increased in response to nutrient starvation in fibroblastic NHDFs and endothelial HUVECs. (G) The expression level of JHDM1D-AS1 is increased in response to nutrient starvation in NHDFs and HUVECs. (H) The expression levels of JHDM1D and JHDM1D-AS1 are increased in the avascular tumor tissues from day 3 to day 5. Data are presented as the mean ± standard error of the mean (SEM) from at least three independent experiments. The expression of each transcript is reported relative to that of β-actin and was determined by real-time quantitative PCR (qPCR) analysis. Student's *t* tests were performed for the indicated comparisons (***, *P* < 0.005; §§§, *P* < 0.005).

We previously reported that JHDM1D regulates tumor growth and angiogenesis under nutrient starvation (11). Using chromatin immunoprecipitation sequencing (ChIP-seq) and formaldehyde-assisted isolation of regulatory element sequencing (FAIRE-seq) in nutrient-starved PANC-1 cells, we found a promoter antisense lncRNA, JHDM1D-AS1, sharing a common promoter that was characterized in the RefSeq database (Fig. 1A) concomitant with the active histone mark of H3K27 acetylation and a nucleosome-free region (Fig. 1A). Expression of JHDM1D and JHDM1D-AS1 was highly correlated in various cancer cell lines (Fig. 1B). The expression of both JHDM1D and JHDM1D-AS1 was decreased by deletion of common 5′ promoter regions of JHDM1D and JHDM1D-AS1 using guide RNA (gRNA)-mediated genome editing (Fig. 1C; Table 1), suggesting that JHDM1D and JHDM1D-AS1 share their promoter. JHDM1D was previously reported to be upregulated under nutrient starvation in various cancer cells (11); therefore, we hypothesized that JHDM1D-AS1 could also be upregulated under starvation. Expression of JHDM1D-AS1 and JHDM1D was commonly increased under nutrient starvation in the PANC-1 and AsPC-1 cell lines (Fig. 1D and E). The expression of JHDM1D and JHDM1D-AS1 was also increased in other cancer cell lines, including HeLa cervical cancer cells, T98G glioblastoma cells, and SW620 colorectal cancer cells (Fig. 1D and E). Expression of JHDM1D and JHDM1D-AS1 was increased in normal cells, including normal human dermal fibroblasts (NHDFs) and human umbilical vein endothelial cells (HUVECs) (Fig. 1F and G). We have previously reported that the mRNAs for nutrient starvation-responsive genes, including JHDM1D, the sense gene of JHDM1D-AS1 that is upregulated under nutrient starvation *in vitro*, were also upregulated *in vivo* in avascular tumor tissues (11, 12) To investigate whether RNA expression of JHDM1D-AS1 is increased in avascular tumor tissues *in vivo*, PANC-1 cells were inoculated in *scid/scid* mice, and tumor samples were obtained on day 0, day 3, day 5, and day 10 (*n* = 3 per each time point). We found that expression of JHDM1D and JHDM1D-AS1 was increased in avascular tumor tissue, especially on day 3 compared to day 5 and day 10 *in vivo* (Fig. 1H). Thus, the nutrient starvation-induced upregulation of JHDM1D and JHDM1D-AS1 may be not specific to pancreatic cancer cells. Together these results suggest that JHDM1D-AS1 may play an essential role in cancer cells.

**TABLE 1:** Promoter sequences deleted by guide RNAs

gRNA set	Deleted sequence
1	CGGCGCGCGCTCCCCGCTCCTCTCCGCGACGGCCGGGCGGAGGGAGCTGTTGAAGGGCACGCAGGCGGCTGCGGGGGCGGAGGGAGCTGGTGGCGGCGGGCGCGCGGCCGCAGCCGGAGGAGGACGGCGGGAGCGTGCGAGGAGCTGGCTCGGTTATTTCGGAGCGAGAGCCGAGGCCGGGGGAAGTTCCTGCGGAGTGCTCAAGGGCAGAAGAGGTGCCGCGTCCCGAAGAGGGGAAGCGGAGAAGTTTGCTGCTGCCCGGGTCGCCT
2	CGGCGCGCGCTCCCCGCTCCTCTCCGCGACGGCCGGGCGGAGGGAGCTGTTGAAGGGCACGCAGGCGGCTGCGGGGGCGGAGGGAGCTGGTGGCGGCGGGCGCGCGGCCGCAGCCGGAGGAGGACGGCGGGAGCGTGCGAGGAGCTGGCTCGGTTATTTCGGAGCGAGAGCCG
3	CGGCCGGGCGGAGGGAGCTGTTGAAGGGCACGCAGGCGGCTGCGGGGGCGGAGGGAGCTGGTGGCGGCGGGCGCGCGGCCGCAGCCGGAGGAGGACGGCGGGAGCGTGCGAGGAGCTGGCTCGGTTATTTCGGAGCGAGAGCCGAGGCCGGGGGAAGTTCCTGCGGAGTGCTCAAGGGCAGAAGAGGTGCCGCGTCCCGAAGAGGGGAAGCGGAGAAGTTTGCTGCTGCCCGGGTCGCCT

### Overexpression of JHDM1D-AS1 increases cell growth *in vitro*.

**FIG 2:**
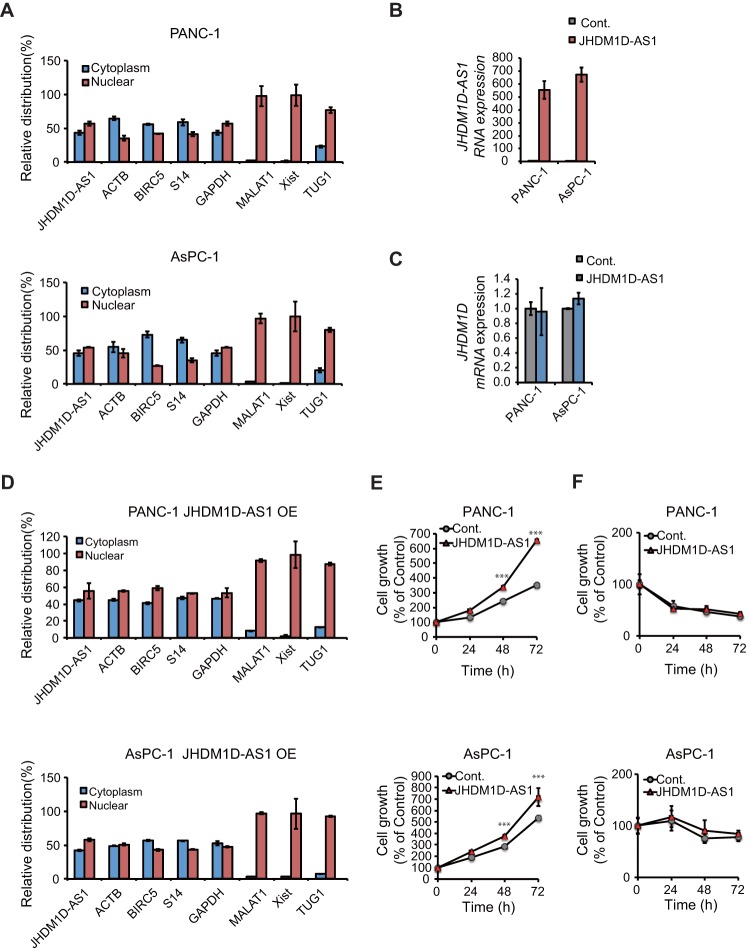
JHDM1D-AS1 is localized in both the cytoplasm and nucleus and increases cell proliferation *in vitro*. (A) Subcellular localization of JHDM1D-AS1. PANC-1 and AsPC-1 cells were subjected to subcellular fractionation, and the amounts of JHDM1D-AS1 in each fraction were evaluated by quantitative real-time PCR. ACTB, BIRC5, S14, and GAPDH genes were used as cytoplasm and nuclear marker genes. MALAT1, Xist, and TUG1 were used as nuclear markers. (B) Expression of JHDM1D-AS1 is stably induced by retroviral transduction of JHDM1D-AS1 in PANC-1 and AsPC-1 cells. (C) The expression level of JHDM1D is not affected by JHDM1D-AS1 overexpression in PANC-1 and AsPC-1 cells. (D) Subcellular localization of JHDM1D-AS1 is not altered by JHDM1D-AS1 overexpression. (E) JHDM1D-AS1 overexpression slightly increased cell growth in control medium in PANC-1 and AsPC-1 cells. (F) JHDM1D-AS1 overexpression does not affect cell growth in nutrient starvation medium in PANC-1 and AsPC-1 cells. Data are presented as the mean ± SEM from at least three independent experiments. The expression of each transcript is reported relative to that of β-actin and was determined by real-time qPCR analysis. Student's *t* tests were performed for the indicated comparisons (***, *P* < 0.005).

JHDM1D-AS1 is an ∼2.4-kbp noncoding RNA without any splicing site, according to the RefSeq database (NR_024451). We examined the cellular localization of JHDM1D-AS1 by subcellular fractionation and reverse transcription-PCR (RT-PCR) of JHDM1D-AS1. We found that JHDM1D-AS1 was localized in both the cytoplasm and nucleus in PANC-1 and AsPC-1 cells, which is similar to the localization of coding mRNAs such as those encoding ACTB and GAPDH (glyceraldehyde-3-phosphate dehydrogenase) (Fig. 2A). Other nucleus-retained lncRNAs, including MALAT1, Xist, and TUG1, showed nucleus-specific localization, as previously reported (13).

To investigate the role of JHDM1D-AS1 in tumor progression, we generated PANC-1 and AsPC-1 cells expressing JHDM1D-AS1 by retroviral transduction. The stable expression of JHDM1D-AS1 did not affect mRNA expression of JHDM1D (Fig. 2B and C). The subcellular localization of the overexpressed JHDM1D-AS1 was similar to that of endogenous JHDM1D-AS1 in both PANC-1 and AsPC-1 cells (Fig. 2D). Overexpression of JHDM1D-AS1 slightly increased cell growth in PANC-1 and AsPC-1 cells under growth-rich conditions (Fig. 2E) but had minor effects on cell growth under nutrient starvation conditions (Fig. 2F) *in vitro*. These results suggest that JHDM1D-AS1 may be involved in the growth of cancer cells.

### Induction of JHDM1D-AS1 significantly increases *in vivo* tumor growth by stimulating tumor angiogenesis and infiltration of CD11b^+^ monocyte/macrophage lineage cells.

**FIG 3:**
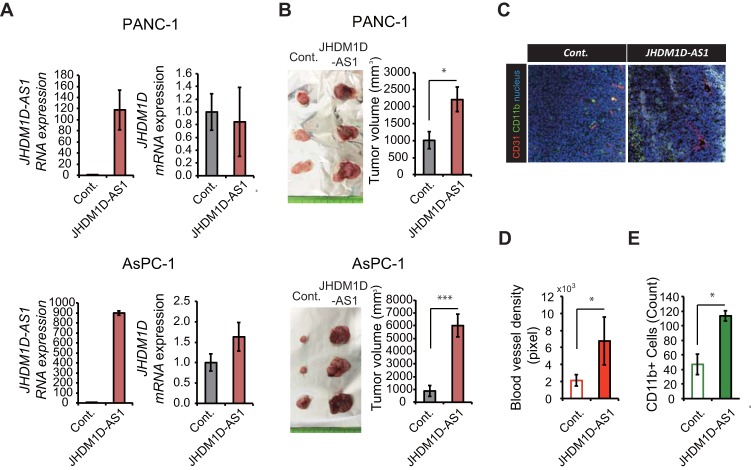
JHDM1D-AS1 overexpression increases tumorigenicity *in vivo* by inducing angiogenesis and macrophage infiltration. (A) Expression of JHDM1D-AS1 is maintained in tumor tissues derived from PANC-1 and AsPC-1 cells stably expressing JHDM1D-AS1 and has only a minor effect on JHDM1D expression in tumor tissues *in vivo*. (B) Overexpression of JHDM1D-AS1 in PANC-1 and AsPC-1 cells accelerates tumor growth *in vivo*. (C) Representative immunostaining images of tumor tissues derived from JHDM1D-AS1-overexpressing AsPC-1 cells compared to those derived from control AsPC-1 cells. (D) Quantitative analysis of CD31^+^ blood vessel density (pixels) in AsPC-1 cells. (E) Quantitative analysis of CD11b^+^ cells (counted-cell number) among AsPC-1 cells. Data are presented as the mean ± SEM from at least three independent experiments. The expression of each transcript is reported relative to that of β-actin and was determined by real-time qPCR analysis. Student's *t* tests were performed for the indicated comparisons (*, *P* < 0.05; ***, *P* < 0.005).

Although JHDM1D-AS1 had minor effects on *in vitro* cell growth, we hypothesized that JHDM1D may play a role in *in vivo* tumor growth (Fig. 2E). To investigate the role of JHDM1D-AS1 in *in vivo* tumor growth, 1 × 10^7^ JHDM1D-AS1-expressing PANC-1 and AsPC-1 cells were subcutaneously inoculated into C.B17/Icr-scidJcl *scid/scid* mice (*n* = 5). We confirmed that JHDM1D-AS1 overexpression was maintained *in vivo* and had no effect on JHDM1D expression (Fig. 3A). Although JHDM1D-AS1 expression occurred at supraphysiological levels in JHDM1D-AS1-overexpressing cells, tumor growth was significantly increased in mice inoculated with both PANC-1-JHDM1D-AS1 and AsPC-1-JHDM1D-AS1 cells compared with that in the control cells (Fig. 3B). To investigate the protumor effect of JHDM1D-AS1 *in vivo*, we examined tumor angiogenesis and infiltration of macrophages within tumor tissues by immunohistochemical analysis (Fig. 3C). Formation of CD31^+^ blood vessels and infiltration of CD11b^+^ monocyte/macrophage lineage cells were significantly increased in tumor tissues (Fig. 3D and E), as reported previously (11, 14), suggesting that JHDM1D-AS1 may increase tumor growth by regulating angiogenesis.

**FIG 4:**
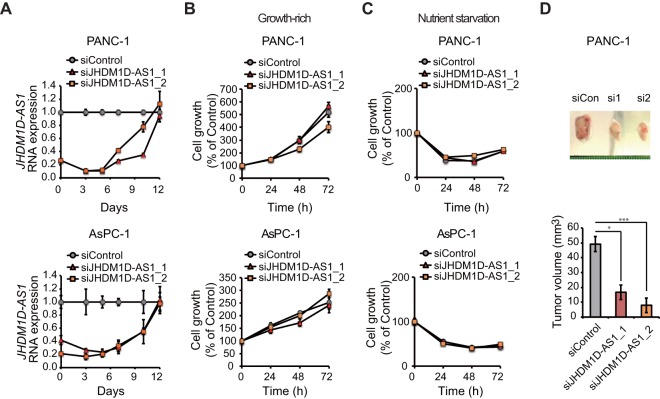
JHDM1D-AS1 knockdown decreased tumorigenicity *in vivo*. (A) Knockdown efficiency of siRNA against JHDM1D-AS1 *in vitro*. (B) JHDM1D-AS1 knockdown does not affect cell growth in the control medium in PANC-1 and AsPC-1 cells. (C) JHDM1D-AS1 knockdown does not affect cell growth under nutrient starvation in PANC-1 and AsPC-1 cells. (D) Depletion of JHDM1D-AS1 by siRNA in PANC-1 and PANC1 cells suppresses tumor growth *in vivo*. The expression of each transcript is reported relative to that of β-actin and was determined by real-time qPCR analysis. Student's *t* tests were performed for the indicated comparisons (*, *P* < 0.05; ***, *P* < 0.005).

To investigate whether silencing of JHDM1D-AS1 small interfering RNAs (siRNAs) influences cancer cell growth *in vitro* and tumor growth *in vivo*, we knocked down JHDM1D-AS1 using siRNA (Fig. 4A). Although inhibition of JHDM1D-AS1 had minor effects on cell growth under growth-rich (Fig. 4B) and nutrient starvation (Fig. 4C) conditions in PANC-1 and AsPC-1 cells *in vitro*, inhibition of JHDM1D-AS1 significantly decreased the tumor growth of PANC-1 cells *in vivo* (Fig. 4D).

### Induction of JHDM1D-AS1 increases angiogenesis by increasing levels of tumor-derived angiogenic factors and host-derived protumorigenic inflammation *in vivo*.

**FIG 5:**
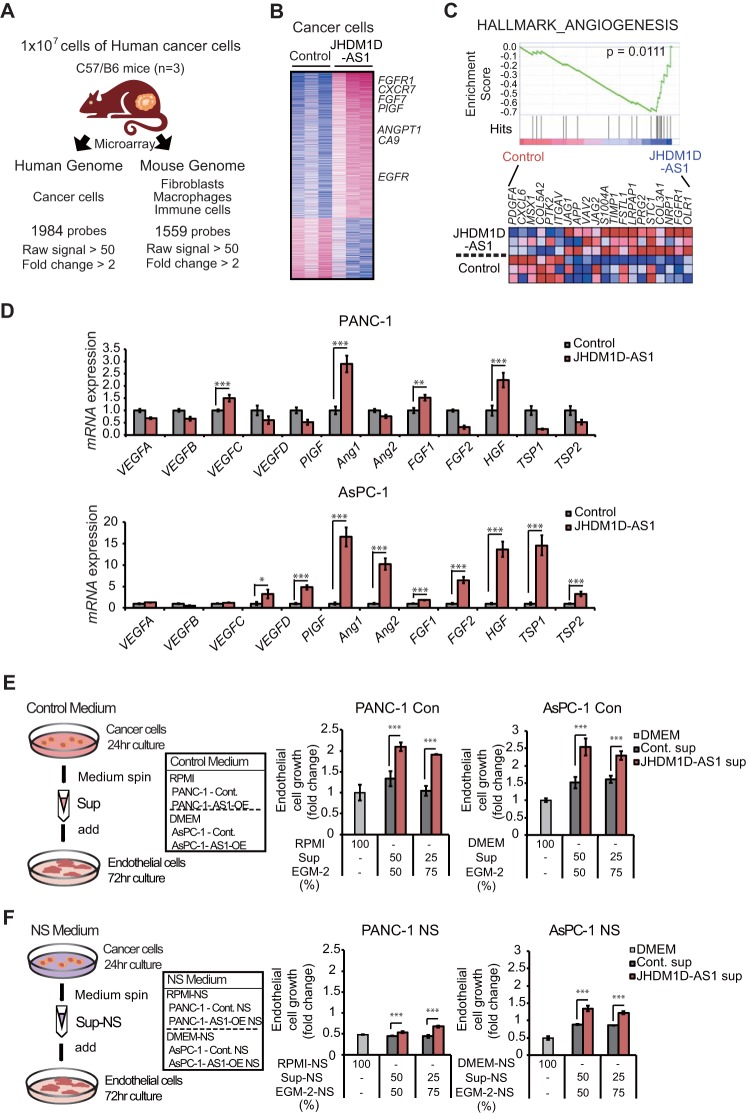
JHDM1D-AS1 overexpression increases the expression of genes for angiogenic factors such as *HGF1* and *FGF1* in cancer cells. (A) Schematic representation of microarray analysis. Expression of mRNA derived from cancer cells and stroma cells was separately examined by using human genome U133 plus 2.0 arrays and mouse genome 430 2.0 arrays, respectively. (B) Heat map representation of 1,983 human cancer cell-derived genes that were up- or downregulated by more than 2-fold *in vivo* in tumor tissues derived from JHDM1D-AS1-overexpressing AsPC-1 cells. (C) Angiogenesis-related genes are upregulated in tumor tissues derived from HDM1D-AS1-overexpressing AsPC-1 cells, as determined by GSEA analysis. (D) Expression of major proangiogenic and antiangiogenic factors in tumor tissues derived from JHDM1D-AS1-expressing PANC-1 and AsPC-1 cells. mRNA expression of proangiogenic and antiangiogenic factors was measured in PANC-1 and AsPC-1 cells by quantitative real-time PCR analysis. (E) Tumor supernatant from JHDM1D-AS1-overexpressing PANC-1 and AsPC-1 cells under the control condition induced endothelial cell growth. The proliferation of human umbilical vein endothelial cells (HUVECs) was measured with a mixture of EGM-2 and either RPMI (for PANC-1 cells) or DMEM (for AsPC-1 cells) culture supernatants. (F) Tumor supernatant from JHDM1D-AS1-overexpressing PANC-1 and AsPC-1 cells under the nutrient-starved condition induced endothelial cell growth. The proliferation of HUVECs was measured with a mixture of EGM-2 and either RPMI-NS (from PANC-1 cells) or DMEM-NS (from AsPC-1 cells) culture supernatants. Data are presented as the mean ± SEM for at least three independent experiments. The expression of each transcript is reported relative to that of β-actin and was determined by real-time qPCR analysis. Student's *t* tests were performed for the indicated comparisons (***, *P* < 0.005).

To investigate whether the angiogenic effects of the JHDM1D-AS1-expressing tumor were derived from regulation of angiogenic factors, mRNA expression in human cancer cells was compared between tumor xenografts comprising hJHDM1D-AS1-expressing AsPC-1 cells and xenografts comprising control AsPC-1 cells by using a human-specific microarray (i.e., Human Genome U133 plus 2.0 oligonucleotide array) (Fig. 5A; see Table S1 in the supplemental material). We found that 1,400 genes were upregulated and 584 genes were downregulated in hJHDM1D-AS1-expressing AsPC-1 xenografts (Fig. 5B). We next conducted a gene set enrichment analysis (GSEA) and found that angiogenic pathways were activated in cancer cells by JHDM1D-AS1 overexpression in tumor tissues *in vivo* (Fig. 5C). We investigated the expression of major pro- and antiangiogenic factors by quantitative real-time PCR and found that human *HGF* and human *FGF1* were significantly upregulated in tumor xenografts comprising hJHDM1D-AS1-expressing PANC-1 cells and those comprising hJHDM1D-AS1-expressing AsPC-1 cells compared with xenografts comprising control cells (Fig. 5D), suggesting that expression of JHDM1D-AS1 increases the expression of angiogenic factors in cancer cells. To investigate the effect of JHDM1D-AS1 on endothelial cell growth, we cultured either control or JHDM1D-AS1-overexpressing pancreatic (PANC-1 and AsPC-1) cells for 24 h under growth-rich and nutrient starvation conditions. Culture supernatants of either control or JHDM1D-AS1-overexpressing cells were concentrated and supplemented to the cell culture of HUVECs *in vitro*. The culture supernatant of JHDM1D-AS1-overexpressing pancreatic (PANC-1 and AsPC-1) cells under growth-rich conditions increased HUVEC growth for 72 h *in vitro* compared to that of empty-vector-treated control cells (Fig. 5E). We additionally examined whether the culture supernatant of JHDM1D-AS1-overexpressing PANC-1 and AsPC-1 cells under starvation could stimulate HUVEC growth under starvation *in vitro*. We found that the culture supernatant of JHDM1D-AS1-overexpressing PANC-1 and AsPC-1 cells under nutrient starvation stimulated endothelial cell growth under nutrient starvation (Fig. 5F), suggesting that JHDM1D-AS1 may stimulate endothelial cell growth under nutrient starvation.

**FIG 6:**
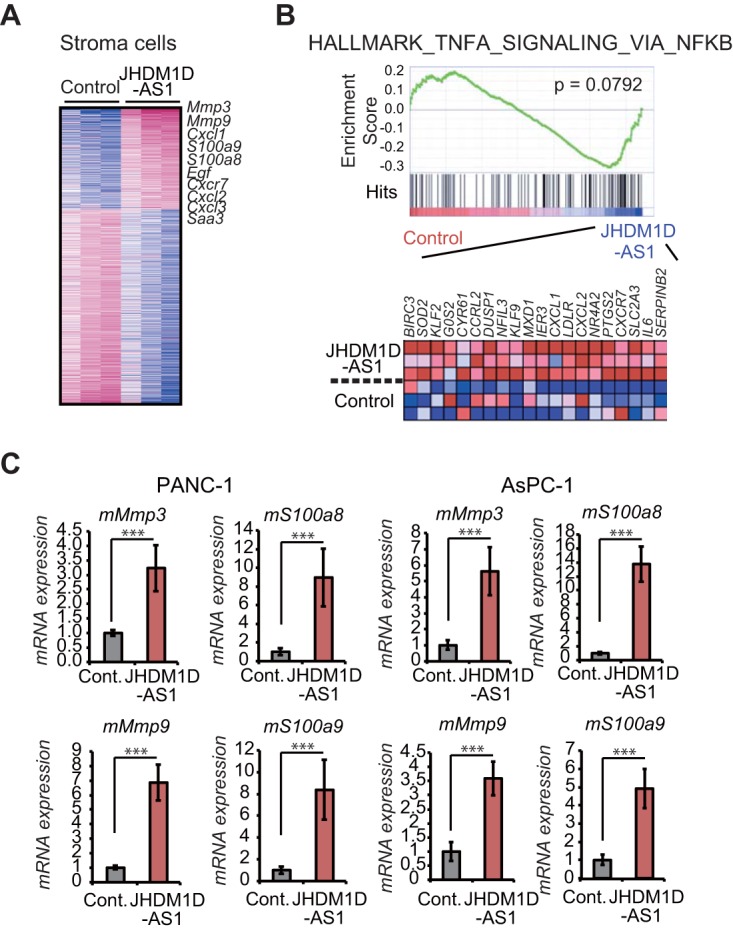
JHDM1D-AS1 overexpression triggers inflammation and upregulation of genes for proinflammatory factors such as *MMP3*, *MMP9*, *S100a8*, and *S100a9* in host-derived cells in tumor tissues. (A) Heat map representation of 1,559 mouse genes that were up- or downregulated by more than 2-fold in tumor tissues derived from JHDM1D-AS1-overexpressing AsPC-1 cells *in vivo*. (B) Mouse genes related to tumor necrosis factor alpha (TNF-α) signaling via NF-κB are upregulated in tumor tissues of mice inoculated with JHDM1D-AS1-expressing AsPC-1 cells. (C) Expression of genes for mouse proinflammatory factors such as m*Mmp3*, m*Mmp9*, m*S100a8*, and m*S100a9* is increased in tumor tissue derived from JHDM1D-AS1-expressing PANC-1 and AsPC-1 cells. Data are presented as the mean ± SEM from at least three independent experiments. The expression of each transcript is reported relative to that of β-actin and was determined by real-time qPCR analysis. Student's *t* tests were performed for the indicated comparisons (***, *P* < 0.005).

We next investigated the expression of host (mouse origin)-derived factors upon JHDM1D-AS1 overexpression (Fig. 6A; see Table S2 in the supplemental material) by using mouse-specific microarrays for tumor xenografts comprising hJHDM1D-AS1-expressing AsPC-1 cells. We identified 1,560 transcripts that were up- or downregulated by more than 2-fold (Fig. 5A and 6A). GSEA analysis indicated that host genes related to NF-κB inflammation signaling were upregulated in tumor tissues of mice inoculated with JHDM1D-AS1-expressing AsPC-1 cells (Fig. 6B). Expression levels of host-derived m*Mmp3*, m*Mmp9*, m*S100a8*, and m*S100a9* were significantly increased in tumor xenografts comprising hJHDM1D-AS1-expressing AsPC-1 and PANC-1 cells compared with those comprising control cells (Fig. 6C). We and others previously reported that expression of host-derived m*Mmp3*, m*Mmp9*, m*S100a8*, and m*S100a9* increases angiogenesis *in vivo* (15, 16). These data suggest that JHDM1D-AS1 promotes angiogenesis by regulating multiple tumor-derived angiogenic factors and host-derived proangiogenic inflammatory genes, resulting in tumor progression.

### JHDM1D-AS1 is highly expressed in tumor tissues and contributes to malignancy.

**FIG 7:**
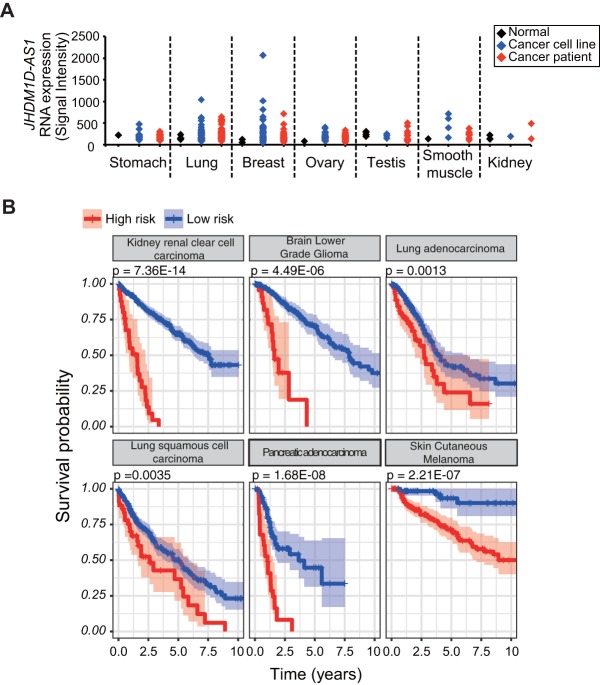
JHDM1D-AS1 is highly expressed in various cancer patients and correlates with poor prognosis. (A) The JHDM1D-AS1 expression level is elevated in various cancer cell lines and clinical samples from cancer patients (the expression data were obtained from Affymetrix Exon array data from our institutional database RefExA [http://www.lsbm.org/site_e/database/index.html]). (B) Kaplan-Meier plots for estimation of overall survival of patients with 330 bladder urothelial carcinoma (BLCA), 236 cervical squamous cell carcinoma and endocervical adenocarcinoma (CESC), 163 glioblastoma multiforme (GBM), 595 kidney renal clear cell carcinoma (KIRC), 365 liver hepatocellular carcinoma (LIHC), 490 lung adenocarcinoma (LUAD), 468 lung squamous cell carcinoma (LUSC), 152 pancreatic adenocarcinoma (PAAD), 290 skin cutaneous melanoma (SKCM), and 344 stomach adenocarcinoma (17) samples. Patients were classified into two groups, high risk and low risk, by the Lasso-regularized Cox proportional-hazard model based on expression profiles of JHDM1D-AS1 direct or indirect target genes (JHDM1D-AS1 signature).

Because JHDM1D-AS1 increased *in vivo* tumor growth in mice (Fig. 3), we hypothesized that the JHDM1D-AS1 may contribute to cancer progression, including overall survival, in patients. We found that JHDM1D-AS1 expression was highly increased in various cancer cell lines and cancer patient samples compared to that in normal cells and tissues (Fig. 7A).

To further evaluate the prognostic effects of JHDM1D-AS1 on overall survival in cancer patients, we constructed a prognostic model based on the expression profiles of JHDM1D-AS1 target genes from 18 cancer cohort studies from The Cancer Genome Atlas (TCGA) (330 bladder urothelial carcinoma [BLCA], 1,073 breast invasive carcinoma [BRCA], 236 cervical squamous cell carcinoma and endocervical adenocarcinoma [CESC], 147 esophageal carcinoma [ESCA], 163 glioblastoma multiforme [GBM], 465 head and neck squamous cell carcinoma [HNSC], 595 kidney renal clear-cell carcinoma [KIRC], 286 kidney renal papillary cell carcinoma [KIRP], 481 brain lower-grade glioma [LGG], 365 liver hepatocellular carcinoma [LIHC], 490 lung adenocarcinoma [LUAD], 468 lung squamous cell carcinoma [LUSC], 302 ovarian serous cystadenocarcinoma [OV], 152 pancreatic adenocarcinoma [PAAD], 208 sarcoma [SARC], 290 skin cutaneous melanoma [SKCM], 344 stomach adenocarcinoma [17], and 376 uterine corpus endometrial carcinoma [UCEC] samples). The JHDM1D-AS1 target genes were identified as differentially expressed genes in JHDM1D-AS1-overexpressed AsPC1 xenografts and a mock control and were called the “JHDM1D-AS1 signature” (see Table S3 in the supplemental material). We used a Lasso-regularized Cox proportional-hazard model to build a JHDM1D-AS1 signature-based prognostic classifier. The results of the Cox model in each cohort are summarized in Table 2. Among the 18 cohort studies, the Lasso using Akaike's information criterion (AICc) selected a null model with just a *y* intercept in 7 studies (BRCA, ESCA, HNSC, KIRP, LGG, OV, and SARC), which were not used in further analysis. For the other 11 cohort studies (BLCA, CESC, GBM, KIRC, LIHC, LUAD, LUSC, PAAD, SKCM, STAD, and UCEC), patients can be classified into two groups (high- and low-risk groups), and the difference in the survival between the two patient groups is significant (*P* value [log rank test] of <0.01 for the 10 cohort studies) (Fig. 7B), and it is also in the other cohorts (Table 3). These results indicated that JHDM1D-AS1 and its target genes were associated with overall survival in patients with several types of cancer.

**TABLE 2:** Summary of the results for the Cox model in each cohort

Tumor	Disease	No. of samples			No. of genes selected by Cox model	*P* value (log rank)
		Total	High risk	Low risk		
KIRC	Kidney renal clear cell carcinoma	595	49	546	25	7.36E−14
PAAD	Pancreatic adenocarcinoma	152	30	122	25	1.68E−08
SKCM	Skin cutaneous melanoma	290	195	95	16	2.21E−07
LIHC	Liver hepatocellular carcinoma	365	270	95	31	3.56E−07
GBM	Glioblastoma multiforme	163	42	121	18	4.49E−06
STAD	Stomach adenocarcinoma	344	282	62	18	8.12E−06
LUAD	Lung adenocarcinoma	490	204	286	8	0.001342361
LUSC	Lung squamous cell carcinoma	468	53	415	7	0.003574324
BLCA	Bladder urothelial carcinoma	330	302	28	19	0.005061063
CESC	Cervical squamous cell carcinoma and endocervical adenocarcinoma	236	178	58	19	0.006150851
UCEC	Uterine corpus endometrial carcinoma	376	341	35	11	0.054558634

**TABLE 3:** Summary of the results of the Cox model in other cohorts

Study	Platform	Cancer type	No. of samples			No. of genes selected by Lasso regularization using AICc	*P* value (log rank)	Corrected *P* value (false-discovery rate)
			Total	High risk	Low risk			
GSE32062	GPL6480	Ovarian	260	51	209	22	2.28E−11	2.62E−10
GSE30219	GPL570	Lung	289	230	59	21	4.04E−09	3.10E−08
GSE1456	GPL97	Breast	159	64	95	8	6.06E−09	3.98E−08
GSE22153	GPL6102	Skin	54	16	38	8	9.41E−09	5.41E−08
GSE22762	GPL97	Heme	30	1	29	6	7.24E−08	3.33E−07
GSE4412	GPL97	Brain	85	1	84	13	2.85E−07	1.09E−06
GSE4271	GPL97	Brain	77	10	67	20	3.41E−07	1.21E−06
GSE14333	GPL570	Colorectal	226	2	224	14	1.44E−06	4.74E−06
GSE4922	GPL96	Breast	242	19	223	8	1.53E−05	4.68E−05
GSE21501	GPL4133	Pancreas	102	67	35	9	3.12E−05	8.96E−05
GSE42669	GPL6244	Brain	55	1	54	2	4.43E−05	0.000119
GSE9195	GPL570	Breast	77	37	40	26	4.64E−05	0.000119
GSE3494	GPL97	Breast	234	185	49	7	7.61E−05	0.000184
GSE8842	GPL5689	Ovarian	82	48	34	30	0.000167	0.000384
GSE17260	GPL6480	Ovarian	110	56	54	2	0.000263	0.000576
GSE37418	GPL570	Brain	73	57	16	4	0.000428	0.000895
GSE1379	GPL1223	Breast	60	35	25	3	0.000843	0.001687
GSE13213	GPL6480	Lung	117	42	75	4	0.001022	0.001923
GSE32063	GPL6480	Ovarian	40	33	7	4	0.001045	0.001923
GSE17710	GPL9053	Lung	56	5	51	3	0.001087	0.001923
GSE9893	GPL5049	Breast	155	18	137	3	0.001171	0.001994
GSE18229	GPL887	Breast	53	1	52	4	0.001946	0.003197
GSE425	GPL317	Breast	22	15	7	6	0.005002	0.007669
GSE13041	GPL8300	Brain	49	46	3	4	0.007634	0.010973
GSE3	GPL10	Adrenal	32	18	14	6	0.015127	0.021087
GSE6532	GPL96	Breast	306	129	177	8	0.017094	0.023127
GSE18229	GPL885	Breast	13	1	12	2	0.019016	0.023769
GSE19234	GPL570	Skin	44	40	4	44	0.141582	0.155066
GSE4412	GPL96	Brain	85	84	1	4	0.280437	0.293184
GSE14814	GPL96	Lung	133	132	1	2	0.401377	0.410296

## DISCUSSION

We have shown that the lncRNA JHDM1D-AS1 is upregulated in response to nutrient starvation and that the subsequent increase in solid pancreatic tumor growth is associated with upregulation of genes for several proangiogenic factors, such as *HGF* and *FGF1*, and proinflammatory factors, such as *MMP*3, *MMP9*, *S100a8*, and *S100a9*, resulting in tumor progression.

Antisense lncRNAs are transcribed from the promoter regions of the coding gene and can be important not only for the expression of sense genes but also for the expression of distant genes under cellular stress, such as hypoxic conditions (18, 19). Here, we identified a long noncoding antisense transcript of JHDM1D, JHDM1D-AS1, whose expression levels increased under nutrient starvation in accordance with the levels of JHDM1D. The high expression of JHDM1D-AS1 in various cancer cell lineages and in patients suggests an important role of this lncRNA in cancer progression.

Many lncRNAs localize mainly in the nuclear fraction, which is critical for their role in chromatin remodeling in *cis* or *trans* (2). In the present study, fractionation analysis revealed that JHDM1D-AS1 is localized in both the cytosol and nucleus, similar to the case for coding mRNAs, suggesting a dual role of JHDM1D-AS1 in not only the nucleus but also the cytoplasm. In addition, JHDM1D-AS1 overexpression did not affect JHDM1D expression, suggesting that JHDM1D-AS1 may not function in *cis*. lncRNAs that are exported to the cytoplasm can interfere with protein posttranscriptional modification, alter gene expression by acting as a decoy for miRNA, and modulate mRNA translation (20). An important goal for future studies should be to determine the mechanisms underlying how JHDM1D-AS1 exerts its role in cancer cell growth.

Because JHDM1D-AS1 accelerated cell growth *in vitro*, we hypothesized that it could accelerate tumor growth *in vivo*. A xenograft study revealed that JHDM1D-AS1 overexpression indeed increased tumor growth *in vivo*, accompanied by elevated blood vessel formation and macrophage infiltration. Although overexpression of JHDM1D-AS1 had minor effects on cell growth *in vitro*, JHDM1D-AS1 overexpression resulted in a significant increase in tumor growth *in vivo*. We previously reported that overexpression and knockdown of the JHDM1D gene, the sense coding gene of JHDM1D-AS1, had minor effects on cell growth under growth-rich and nutrient starvation conditions *in vitro* but induced significant tumor growth *in vivo* by regulating angiogenesis (11). Thus, it is conceivable that JHDM1D-AS1 may also influence the tumor microenvironment rather than the cancer cells themselves.

Angiogenesis is orchestrated by various proangiogenic factors, such as VEGFs, FGFs, HGF, and the angiopoietin/Tie system, in addition to many other growth factors (21, 22). In the present study, microarray analysis and subsequent quantitative PCR revealed that JHDM1D-AS1-overexpressing AsPC-1 and PANC-1 cancer cells showed increased angiogenesis through upregulation of genes for angiogenic factors, including *HGF* and *FGF1*. We and others previously demonstrated that the nutrient-deprived tumor microenvironment and several long noncoding RNAs can promote tumor angiogenesis (11, 19, 23–25), but the specific lncRNAs involved in angiogenesis under nutrient starvation are not fully understood. To the best of our knowledge, this is the first report suggesting that an lncRNA responsive to nutrient starvation plays an important role in angiogenesis by modulating multiple angiogenic factors.

Interactions between cancer cells and the associated stroma as well as immune cells such as macrophages contribute to tumor angiogenesis and progression (26). In the present study, xenografting of JHDM1D-AS1-overexpressing cancer cells triggered inflammation in the surrounding host stromal cells by upregulation of inflammatory genes such as *Mmp3*, *Mmp9*, *S100a8*, and *S100a9*. Tumor-promoting inflammation is one of the hallmarks of cancer progression; thus, these data may explain why JHDM1D-AS1 overexpression had more impact on *in vivo* tumor growth than on *in vitro* cell proliferation. We and others have previously reported that infiltration of macrophage lineage cells associated with increased levels of host-derived MMP3, MMP9, S100A8, and S100A9 stimulated angiogenesis (11, 14, 27). Thus, JHDM1D-AS1 may promote tumor angiogenesis by secretion of proangiogenic factors and by stimulation of proangiogenic inflammation.

To identify the JHDM1D-AS1 direct or indirect target genes called the “JHDM1D-AS1 signature,” we performed DNA microarray analysis of JHDM1D-AS1-overexpressed AsPC1 xenografts (case) and a mock control. As a result, a total of 237 probes corresponding to 150 genes were selected as differentially expressed genes in JHDM1D-AS1-overexpressing tumor tissues and used in further analysis. To assess the impact of JHDM1D-AS1-associated genes on prognosis in cancer patients, we conducted survival analysis using the Lasso-regularized Cox proportional-hazard model based on the expression profiles of the JHDM1D-AS1 signature in patients with various types of cancer. Remarkably, the prognostic model of JHDM1D-AS1-associated genes was classified into two groups (groups with worse and better prognosis), further supporting the clinical importance of JHDM1D-AS1.

In conclusion, our findings provide evidence that JHDM1D-AS1 is upregulated under nutrient starvation in the tumor microenvironment and accelerates tumor angiogenesis and growth *in vivo* by modulating expression of genes for angiogenic factors such as *HGF* and *FGF1* and triggering inflammation in the surrounding tumor microenvironment, thereby promoting malignancy of cancer. Based on these results, we suggest that inhibition of the nutrient starvation-responsive lncRNA JHDM1D-AS1 can be a potential therapeutic approach for cancer in the future.

## MATERIALS AND METHODS

### Cell culture, hypoxia, and nutrient starvation.

The human pancreatic carcinoma epithelial-like cell line PANC-1 and the adenocarcinoma ascites metastasis cell line AsPC-1 were purchased from the American Type Culture Collection (Manassas, VA). Cells were maintained in RPMI 1640 medium (Nacalai Tesque, Kyoto, Japan) and Dulbecco's modified Eagle's medium (DMEM) (Nacalai Tesque, Kyoto, Japan) supplemented with 10% fetal bovine serum (FBS) at 37°C in a 5% CO_2_ atmosphere, respectively. Normal human dermal fibroblasts (NHDFs) and human umbilical vein endothelial cells (HUVECs) were purchased from Lonza (Tokyo, Japan) and maintained in fibroblast basal medium (FBM) or endothelial cell basal medium 2 (EBM2) (Lonza, Tokyo, Japan) at 37°C in a 5% CO_2_ atmosphere, respectively.

Nutrient deprivation medium was prepared to contain inorganic salts, i.e., 0.2 g/liter CaCl_2_ (anhydrous), 0.1 mg/liter Fe(NO_3_)_3_/9H_2_O, 0.4 g/liter KCl, 97.67 mg/liter MgSO_4_ (anhydrous), 6.4 g/liter NaCl, 3.7 g/liter NaHCO_3_, 0.125 g/liter NaH_2_PO_4_/H_2_O, and 15 mg/liter phenol red, according to the composition of DMEM. Then, 1% nutrient-containing medium was prepared from a mixture of the nutrient deprivation medium and the complete nutrient DMEM supplemented with 10% FBS at a ratio of 99:1. Cells were seeded in complete fresh medium for 20 h. The cells were then washed with phosphate-buffered saline (PBS), nutrient starvation was initiated by replacement with nutrient-deprived medium containing 1% nutrition, and cells were cultured for 24 h, unless otherwise stated, as previously described (11, 28).

### Cell proliferation assay.

Cell proliferation was measured using the sulforhodamine B (SRB) assay as previously described (28).

### Fractionation.

Cells (∼1 × 10^7^) were collected using a cell scraper and centrifuged at 500 × *g* for 5 min. The cell pellet was washed in ice-cold RSB150 buffer (10 mM Tris-HCl [pH 7.4], 150 mM NaCl, 2.5 mM MgCl_2_) and centrifuged. The cell pellet was resuspended in 400 μl of ice-cold RSB150 buffer, and then 0.25 mg/ml digitonin was added to the cells and incubated for 5 min on ice. The cells were centrifuged at 3,000 × *g* for 1 min at 4°C, yielding the cytoplasmic (supernatant) and nuclear (pellet) fractions. The supernatant was reserved on ice as a source of the cytoplasmic fraction. The nuclear pellet was washed twice in ice-cold RSB150 buffer and centrifuged. The nuclear pellet was resuspended in 400 μl of ice-cold RSB150 buffer and 0.5% Triton X-100. The obtained fractions were subjected to RNA extraction using TRIzol LS (Thermo Fisher Scientific, MA) in accordance with the manufacturer's instructions. Samples were eluted with 40 μl of RNase-free water for each fraction.

### Gene expression analysis using real-time PCR.

Total RNA was extracted from cells using the Isogen reagent (Nippon Gene, Toyama, Japan), converted to cDNA by using the Prime Script reverse transcriptase (TaKaRa, Shiga, Japan) as per the manufacturer's instructions, and used for quantitative real-time PCR amplification with SYBR green (TaKaRa) (Table 4).

**TABLE 4:** qRT-PCR primers used in this study

Primer	Forward sequence	Reverse sequence
Human		
*ACTB*	5′-AGAAGGAGATCACTGCCCTGGCACC-3′	5′-CCTGCTTGCTGATCCACATCTGCTG-3′
*GAPDH*	5′-ACTTTGTCAAGCTCATTTCCTG-3′	5′-CTCTCTTCCTCTTGGCTCTTG-3′
*BIRC5*	5′-GACCACCGCATCTCTACATTC-3′	5′-CCAAGTCTGGCTCGTTCTC-3′
*S16*	5′-CCAAGTCTGGCTCGTTCTC-3′	5′-CAGGTCCAGGGGTCTTGGTCC-3′
*MALAT1*	5′-CAGGTCCAGGGGTCTTGGTCC-3′	5′-ATTCGGGGCTCTGTAGTCCT-3′
*Xist*	5′-CCCGTCTTTTGTTGGACAGT-3′	5′-GGATTCTCCAGAAGCACAGC-3′
*TUG1*	5′-CAAGCACTACCACCAGCACTGTTAC-3′	5′-GCAATCAGGAGGCACAGGACATAAT-3′
*VEGF-A*	5′-GCGGAGAAAGCATTTGTTTGT-3′	5′-CGGCTTGTCACATCTGCAAC-3′
*VEGF-B*	5′-GATCCGGTACCCGAGCAGTCAG-3′	5′-CACCTGCAGGTGTCTGGGTTGA-3′
*VEGF-C*	5′-CAGTGTCAGGCAGCGAACAA-3′	5′-CTTCCTGAGCCAGGCATCTG-3′
*VEGF-D*	5′-ATCTGTATGAACACCAGCACCTC-3′	5′-TGGCAACTTTAACAGGCACTAAT-3′
*PlGF*	5′-CCGGCTCGTGTATTTATTACCG-3′	5′-GGCAACCACTGTTCTCCAGAGC-3′
*ANG1*	5′-CCTTCCAGCAATAAGTGGTAGTT-3′	5′-CAAACGGCTCCAGATTCA-3′
*ANG2*	5′-GAGGCTGAGAATCAGACTGACA-3′	5′-TTACTGATAAACTTGCACATAACATTCT-3′
*FGF1*	5′-CAGGGTCCTGGTCCTAAAGAG-3′	5′-TGCCTGAATGCTCAGGTAGAC-3′
*FGF2*	5′-TGAATCACTAACTGACTGAAAATTG-3′	5′-GAAGGGTCTCCCGCATACT-3′
*HGF*	5′-GGTGGCCCACTTGTTTGT-3′	5′-CATCCACGACCAGGAACA-3′
*TSP-1*	5′-CTCTACCAGTGTCCTCCTCACC-3′	5′-TTGTGGCCAATGTAGTTAGTGC-3′
*TSP-2*	5′-AGTTTGGGTCTGTGGACTTCAG-3′	5′-TTCCACATCACCACATAGAAGC-3′
*JHDM1D*	5′-TATTCAGGGCATGCTGTCTATG-3′	5′-GGGATCCTGGAGAGAGTTTCTT-3′
*JHDM1-AS1*	5′-TTGGAGTCTGGCTAAAGAGCA-3′	5′-CTGGGCTTCCTTCTTCATACC-3′
Mouse		
*Actb*	5′-TGACAGGATGCAGAAGGAGA-3′	5′-GCTGGAAGGTGGACAGTGAG-3′
*Mmp3*	5′-TCAGTGGATCTTCGCAGTTG-3′	5′-AGGATGCCTTCCTTGGATCT-3′
*Mmp9*	5′-AGACGACATAGACGGCATCC-3′	5′-GTGGTTCAGTTGTGGTGGTG-3′
*S100a8*	5′-CCTTTGTCAGCTCCGTCTTC-3′	5′-CAAGGCCTTCTCCAGTTCAG-3′
*S100a9*	5′-TCATCGACACCTTCCATCAA-3′	5′-AAAGGTTGCCAACTGTGCTT-3′

### Gene expression profiling and statistical analysis.

For genome-wide transcription analysis, data were collected and analyzed with a GeneChip Scanner 3000 (Affymetrix, CA). The GeneChip data were analyzed using the Affymetrix GeneChip operating software v1.3 with MAS5 algorithms to obtain a signal value (GeneChip score) for each probe set. For global normalization, the average signal in an array was made equal to 100.

### RNA-seq.

RNA-seq reads were aligned to human transcriptome (UCSC gene) and genome (GRCh37/hg19) references using Burrows-Wheeler Aligner. After a transcript coordinate was converted to a genomic positions, an optimal mapping result was selected from either transcript or genome mapping by comparing the minimal edit distance to the reference. Local realignment was performed within an in-house short-reads aligner with a smaller *k*-mer size (*k* = 11). Finally, values for fragments per kilobase of exon per million fragments mapped (FPKM) were calculated for each UCSC gene while considering strand-specific information.

### ChIP.

PANC-1 cells (1 × 10^7^) were cross-linked with 1% formaldehyde for 10 min at room temperature. Fixation was stopped by adding 0.125 M glycine. Nuclear pellets were prepared for chromatin immunoprecipitation (ChIP) as described previously (29). Prewashed magnetic Dynabeads (Life Technologies, MA) were incubated with anti-H3K27ac antibody (Millipore, MA) or anti-SREBP2 antibody (Cayman, MI) in ChIP dilution buffer (20 mM Tris-HCl [pH 8.0], 150 mM NaCl, 1 mM EDTA, 1% Triton X-100, protease inhibitor cocktail [Roche, Basel, Switzerland]) for 6 h by wheel rotating at 4°C. Subsequently, sonicated cross-linked nuclear lysates were added and incubated overnight at 4°C by wheel rotating. The beads were washed several times and eluted with elution buffer (1% SDS and 100 mM NaHCO_3_), and the eluent was incubated with pronase (1 mg ml^−1^) at 42°C for 2 h and then incubated at 65°C overnight. DNA was purified using the QIAquick PCR purification kit (Qiagen, Hilden, Germany). ChIP-seq was done with an Illumina/Solexa sequencer as previously described (29).

### ChIP-seq data processing.

All bound DNA fragments were mapped to the UCSC build hg19 (NCBI build 37) assembly of the mouse genome by using the mapping program ELAND (http://support.illumina.com/sequencing/sequencing_software/casava.html) based on the 50-side 36-bp sequences.

### Formaldehyde-assisted isolation of regulatory elements.

Formaldehyde-assisted isolation of regulatory elements was performed as described previously (30). Briefly, formalin-cross-linked cell pellets were lysed in SDS lysis buffer (50 mM Tris-HCl [pH 8.0], 10 mM ETDA, 1% SDS, and proteinase inhibitor cocktail [Nacalai Tesque, Kyoto, Japan]) and sonicated with an ultrasound homogenizer (Bioruptor UCD-200TM; Cosmo Bio). Samples were subjected to phenol-chloroform extraction twice followed by ethanol precipitation and subsequently were purified using the QIAquick PCR purification kit (Qiagen, Hilden, Germany).

### gRNAs.

Lentivirus CRISPR/Cas9 vectors targeting transcription start site (TSS) regions of JHDM1D-AS1 were constructed using lentiCRISPR v2 vectors (Addgene). Lentiviral particles containing gRNAs were produced using packaging (HEK293Ta) cells according to the manufacturer's instructions (Addgene). Cells were infected with the gRNA lentiviruses with 8 μg/ml Polybrene and subsequently selected using 2 μg/ml puromycin for forward gRNAs and 6 μg/ml blasticidin for reverse gRNAs (Table 5).

**TABLE 5:** Guide RNAs used in this study

Primer	Targeting type	Forward sequence	Reverse sequence
Control	Not applicable	5′-GTCCCCTCCACCCCACAGTG-3′	5′-CACTGTGGGGTGGAGGGGAC-3′
gRNA set 1	Promoter + exon	5′-AGAAGGAGATCACTGCCCTGGCACC-3′	5′-CCTGCTTGCTGATCCACATCTGCTG-3′
gRNA set 2	Promoter + exon	5′-AGAAGGAGATCACTGCCCTGGCACC-3′	5′-TTATTTCGGAGCGAGAGCCG-3′
gRNA set 3	Promoter + exon	5′-CTCCCCGCTCCTCTCCGCGA-3′	5′-TTATTTCGGAGCGAGAGCCG-3′

### Retroviral transfection of JHDM1D-AS1.

A PMX-puro retroviral vector containing full-length human JHDM1D-AS1 was transfected by the pantropic platinum-GP retroviral packaging cell line (PlatGP) (31) using Fugene 6 transfection reagent (Roche). Target cells were infected overnight with the vesicular stomatitis virus (VSV)/Polybrene-containing supernatant. After infection, the colonies were selected in a medium containing 1.5 μg/ml puromycin.

### Animal studies, tumor xenograft, and clinical samples.

Human PANC-1 and AsPC-1 cells (1 × 10^7^) were subcutaneously injected into Icr-scidJcl *scid/scid* mice. The tumor volume was measured, and the data were analyzed using Student's *t* test. All animal care procedures and patient experiments were in accordance with institutional guidelines approved by the University of Tokyo.

### Immunofluorescence staining.

Freshly frozen or 4% paraformaldehyde (PFA)-fixed tumor tissues were cut 14 μm thick by using a Cryostat (Leica, Wetzlar, Germany) and stained with hamster anti-CD31 (BD Biosciences, San Jose, CA) and rat anti-CD11b (BD Biosciences). The sections were incubated with the appropriate secondary antibody and the nucleus-staining dye To-Pro-3 (Invitrogen Life Technologies, Tokyo, Japan) and then analyzed using a confocal microscope (Radiance 2000; Bio-Rad, CA). We quantified the number of CD31^+^ blood vessels/pericytes in the total area of the tumor. We analyzed 5 to 10 fields per sample (*n* = 5) for quantification of blood vessel density (*k* pixels). We also counted the CD11b^+^ cells in the total area of the tumor in 5 to 10 fields for each sample (*n* = 5).

### siRNA.

siRNAs designed against human JHDM1D-AS1 were obtained commercially (Invitrogen Life Technologies, MA). In this study, two sequences of siRNAs were used against JHDM1D-AS1: siJHDM1D-AS1_1 (sense, 5′-GAACCCUUCCGAUUCCCAATT-3′; antisense, 5′-UUGGGAAUCGGAAGGGUUCAT-3′) and siJHDM1D-AS1_2 (sense, 5′-GCAUAUAUAUAGCGGGAAATT-3′; antisense, 5′-UUUCCCGCUAUAUAUAUGCCA-3′). Cells were transfected with JHDM1D-AS1 siRNAs or negative (scramble) control siRNA using Lipofectamine RNAi MAX transfection reagent according to the instructions of the manufacturer (Invitrogen Life Technologies). The ability of the siRNA to inhibit target gene expression was accessed 48 h posttransfection.

### Survival analysis using the JHDM1D-AS1 signature.

By analyzing DNA microarray gene expression profiles of JHDM1D-AS1-overexpressed AsPC1 xenografts (case) and a mock control, a total of 237 probes corresponding to 150 genes were selected, whose average expression level in the case was upregulated more than 4 times compared to that in the control and upregulated more than 75th percentile in that in the case (see Table S3 in the supplemental material). The selected genes were considered direct or indirect target genes of JHDM1D-AS1 and called the “JHDM1D-AS1 signature.” To construct the prognostic model for cancer patients based on the expression profiles of the JHDM1D-AS1 signature, we downloaded normalized RNA-seq expression data sets for 18 cancer cohort studies (330 bladder urothelial carcinoma [BLCA], 1,073 breast invasive carcinoma [BRCA], 236 cervical squamous cell carcinoma and endocervical adenocarcinoma [CESC], 147 esophageal carcinoma [ESCA], 163 glioblastoma multiforme [GBM], 465 head and neck squamous cell carcinoma [HNSC], 595 kidney renal clear-cell carcinoma [KIRC], 286 kidney renal papillary cell carcinoma [KIRP], 481 brain lower-grade glioma [LGG], 365 liver hepatocellular carcinoma [LIHC], 490 lung adenocarcinoma [LUAD], 468 lung squamous cell carcinoma [LUSC], 302 ovarian serous cystadenocarcinoma [OV], 152 pancreatic adenocarcinoma [PAAD], 208 sarcoma [SARC], 290 skin cutaneous melanoma [SKCM], 344 stomach adenocarcinoma [17], and 376 uterine corpus endometrial carcinoma [UCEC] samples) from the Broad TCGA GDAC website (http://gdac.broadinstitute.org/). We then used a Lasso-regularized Cox proportional-hazard model with the glmnet package (version 2.0-5) (32) in the R statistical environment (version 3.3.2) to build a JHDM1D-AS1 signature-based prognostic classifier. The tuning parameter λ in the Lasso regularization was chosen by using the corrected Akaike's information criterion (AICc) (33, 34). The results of the Cox model in each cohort are summarized in Table 2. Among the 18 cohort studies, the Lasso using AICc selected a null model with just an *y* intercept in 7 studies (BRCA, ESCA, HNSC, KIRP, LGG, OV, and SARC), which were not used in further analysis. For the other 11 cohort studies (BLCA, CESC, GBM, KIRC, LIHC, LUAD, LUSC, PAAD, SKCM, STAD, and UCEC), patients were classified into two groups based on whether the risk score in the Cox model was more than 0 (high risk and worse prognosis) or less than 0 (low risk and better prognosis). To evaluate the prognostic significance of the Cox model, we used the Kaplan-Meier method, and the *P* value was calculated using the log rank test. For adjustment for multiple hypothesis testing, the *P* values were corrected by the Benjamini-Hochberg procedure (35).

## Supplementary Material

Supplemental material

## References

[1] Djebali S, Davis CA, Merkel A, Dobin A, Lassmann T, Mortazavi A, Tanzer A, Lagarde J, Lin W, Schlesinger F, Xue C, Marinov GK, Khatun J, Williams BA, Zaleski C, Rozowsky J, Roder M, Kokocinski F, Abdelhamid RF, Alioto T, Antoshechkin I, Baer MT, Bar NS, Batut P, Bell K, Bell I, Chakrabortty S, Chen X, Chrast J, Curado J, Derrien T, Drenkow J, Dumais E, Dumais J, Duttagupta R, Falconnet E, Fastuca M, Fejes-Toth K, Ferreira P, Foissac S, Fullwood MJ, Gao H, Gonzalez D, Gordon A, Gunawardena H, Howald C, Jha S, Johnson R, Kapranov P, King B, 2012 Landscape of transcription in human cells. Nature 489:101–108. doi:10.1038/nature11233. 22955620 PMC3684276

[2] Fatica A, Bozzoni I 2014 Long non-coding RNAs: new players in cell differentiation and development. Nat Rev Genet 15:7–21. doi:10.1038/nri3777. 24296535

[3] Mercer TR, Dinger ME, Mattick JS 2009 Long non-coding RNAs: insights into functions. Nat Rev Genet 10:155–159. doi:10.1038/nrg2521. 19188922

[4] Wilusz JE, Sunwoo H, Spector DL 2009 Long noncoding RNAs: functional surprises from the RNA world. Genes Dev 23:1494–1504. doi:10.1101/gad.1800909. 19571179 PMC3152381

[5] Amaral PP, Dinger ME, Mattick JS 2013 Non-coding RNAs in homeostasis, disease and stress responses: an evolutionary perspective. Brief Funct Genomics 12:254–278. doi:10.1093/bfgp/elt016. 23709461

[6] Carmeliet P 2003 Angiogenesis in health and disease. Nat Med 9:653–660. doi:10.1038/nm0603-653. 12778163

[7] Ferrara N, Kerbel RS 2005 Angiogenesis as a therapeutic target. Nature 438:967–974. doi:10.1038/nature04483. 16355214

[8] Ono M 2008 Molecular links between tumor angiogenesis and inflammation: inflammatory stimuli of macrophages and cancer cells as targets for therapeutic strategy. Cancer Sci 99:1501–1506. doi:10.1111/j.1349-7006.2008.00853.x. 18754859 PMC11158258

[9] Conway EM, Collen D, Carmeliet P 2001 Molecular mechanisms of blood vessel growth. Cardiovasc Res 49:507–521. doi:10.1016/S0008-6363(00)00281-9. 11166264

[10] Folkman J, Klagsbrun M 1987 Angiogenic factors. Science 235:442–447. doi:10.1126/science.2432664. 2432664

[11] Osawa T, Muramatsu M, Wang F, Tsuchida R, Kodama T, Minami T, Shibuya M 2011 Increased expression of histone demethylase JHDM1D under nutrient starvation suppresses tumor growth via down-regulating angiogenesis. Proc Natl Acad Sci U S A 108:20725–20729. doi:10.1073/pnas.1108462109. 22143793 PMC3251107

[12] Osawa T, Tsuchida R, Muramatsu M, Shimamura T, Wang F, Suehiro J, Kanki Y, Wada Y, Yuasa Y, Aburatani H, Miyano S, Minami T, Kodama T, Shibuya M 2013 Inhibition of histone demethylase JMJD1A improves anti-angiogenic therapy and reduces tumor-associated macrophages. Cancer Res 73:3019–3028. doi:10.1158/0008-5472.CAN-12-3231. 23492365

[13] Cabili MN, Dunagin MC, McClanahan PD, Biaesch A, Padovan-Merhar O, Regev A, Rinn JL, Raj A 2015 Localization and abundance analysis of human lncRNAs at single-cell and single-molecule resolution. Genome Biol 16:20. doi:10.1186/s13059-015-0586-4. 25630241 PMC4369099

[14] Muramatsu M, Yamamoto S, Osawa T, Shibuya M 2010 Vascular endothelial growth factor receptor-1 signaling promotes mobilization of macrophage lineage cells from bone marrow and stimulates solid tumor growth. Cancer Res 70:8211–8221. doi:10.1158/0008-5472.CAN-10-0202. 20924106

[15] Kessenbrock K, Plaks V, Werb Z 2010 Matrix metalloproteinases: regulators of the tumor microenvironment. Cell 141:52–67. doi:10.1016/j.cell.2010.03.015. 20371345 PMC2862057

[16] Hiratsuka S, Watanabe A, Aburatani H, Maru Y 2006 Tumour-mediated upregulation of chemoattractants and recruitment of myeloid cells predetermines lung metastasis. Nat Cell Biol 8:1369–1375. doi:10.1038/ncb1507. 17128264

[17] Rofstad EK, Mathiesen B, Kindem K, Galappathi K 2006 Acidic extracellular pH promotes experimental metastasis of human melanoma cells in athymic nude mice. Cancer Res 66:6699–6707. doi:10.1158/0008-5472.CAN-06-0983. 16818644

[18] Yang F, Bi J, Xue X, Zheng L, Zhi K, Hua J, Fang G 2012 Up-regulated long non-coding RNA H19 contributes to proliferation of gastric cancer cells. FEBS J 279:3159–3165. doi:10.1111/j.1742-4658.2012.08694.x. 22776265

[19] Tee AE, Liu B, Song R, Li J, Pasquier E, Cheung BB, Jiang C, Marshall GM, Haber M, Norris MD, Fletcher JI, Dinger ME, Liu T 2016 The long noncoding RNA MALAT1 promotes tumor-driven angiogenesis by up-regulating pro-angiogenic gene expression. Oncotarget 7:8663–8675. doi:10.18632/oncotarget.6675. 26848616 PMC4890995

[20] Wang KC, Chang HY 2011 Molecular mechanisms of long noncoding RNAs. Mol Cell 43:904–914. doi:10.1016/j.molcel.2011.08.018. 21925379 PMC3199020

[21] Shibuya M 2008 Vascular endothelial growth factor-dependent and-independent regulation of angiogenesis. BMB Rep 41:278–286. doi:10.5483/BMBRep.2008.41.4.278. 18452647

[22] Distler JH, Hirth A, Kurowska-Stolarska M, Gay RE, Gay S, Distler O 2003 Angiogenic and angiostatic factors in the molecular control of angiogenesis. Q J Nucl Med 47:149–161. 12897707

[23] Wang Y, Ning Y, Alam GN, Jankowski BM, Dong Z, Nör JE, Polverini PJ 2013 Amino acid deprivation promotes tumor angiogenesis through the GCN2/ATF4 pathway. Neoplasia 15:989–997. doi:10.1593/neo.13262. 23908598 PMC3730049

[24] Cai H, Liu X, Zheng J, Xue Y, Ma J, Li Z, Xi Z, Li Z, Bao M, Liu Y 2017 Long non-coding RNA taurine upregulated 1 enhances tumor-induced angiogenesis through inhibiting microRNA-299 in human glioblastoma. Oncogene 36:318–331. doi:10.1038/onc.2016.212. 27345398

[25] Lu Z, Xiao Z, Liu F, Cui M, Li W, Yang Z, Li J, Ye L, Zhang X 2016 Long non-coding RNA HULC promotes tumor angiogenesis in liver cancer by up-regulating sphingosine kinase 1 (SPHK1). Oncotarget 7:241–254. doi:10.18632/oncotarget.6280. 26540633 PMC4807995

[26] Kalluri R, Zeisberg M 2006 Fibroblasts in cancer. Nat Rev Cancer 6:392–401. doi:10.1038/nrc1877. 16572188

[27] Hiratsuka S, Nakamura K, Iwai S, Murakami M, Itoh T, Kijima H, Shipley JM, Senior RM, Shibuya M 2002 MMP9 induction by vascular endothelial growth factor receptor-1 is involved in lung-specific metastasis. Cancer Cell 2:289–300. doi:10.1016/S1535-6108(02)00153-8. 12398893

[28] Osawa T, Muramatsu M, Watanabe M, Shibuya M 2009 Hypoxia and low-nutrition double stress induces aggressiveness in a murine model of melanoma. Cancer Sci 100:844–851. doi:10.1111/j.1349-7006.2009.01105.x. 19220297 PMC11159247

[29] Matsumura Y, Nakaki R, Inagaki T, Yoshida A, Kano Y, Kimura H, Tanaka T, Tsutsumi S, Nakao M, Doi T, Fukami K, Osborne TF, Kodama T, Aburatani H, Sakai J 2015 H3K4/H3K9me3 bivalent chromatin domains targeted by lineage-specific DNA methylation pauses adipocyte differentiation. Mol Cell 60:584–596. doi:10.1016/j.molcel.2015.10.025. 26590716

[30] Giresi PG, Kim J, McDaniell RM, Iyer VR, Lieb JD 2007 FAIRE (formaldehyde-assisted isolation of regulatory elements) isolates active regulatory elements from human chromatin. Genome Res 17:877–885. doi:10.1101/gr.5533506. 17179217 PMC1891346

[31] Morita S, Kojima T, Kitamura T 2000 Plat-E: an efficient and stable system for transient packaging of retroviruses. Gene Ther 7:1063–1066. doi:10.1038/sj.gt.3301206. 10871756

[32] Simon N, Friedman JH, Hastie T, Tibshirani R 2011 Regularization paths for Cox's proportional hazards model via coordinate descent. 39:13. 10.18637/jss.v039.i05 PMC4824408 27065756

[33] Hurvich CM, Tsai C-L 1989 Regression and time series model selection in small samples. Biometrika 76:297–307. doi:10.1093/biomet/76.2.297.

[34] Sugiura N 1978 Further analysts of the data by Akaike's information criterion and the finite corrections. Commun Stat Theor Methods 7:13–26. doi:10.1080/03610927808827599.

[35] Benjamini Y, Hochberg Y 1995 Controlling the false discovery rate: a practical and powerful approach to multiple testing. J R Stat Soc Ser B 57:289–300.

