# Supplementary material for: Long Noncoding RNA JHDM1D-AS1 Promotes Tumor Growth by Regulating Angiogenesis in Response to Nutrient Starvation
Source: Mol Cell Biol. 2017 Aug 28;37(18):e00125-17. doi: 10.1128/MCB.00125-17 (PMC5574049; doi:10.1128/MCB.00125-17)
Supplement: Supplemental material [file supp_37_18_e00125-17__index.html]

Supplemental material 

# Long Noncoding RNA JHDM1D-AS1 Promotes Tumor Growth by Regulating Angiogenesis in Response to Nutrient Starvation

## Supplemental material

- Supplemental file 1 -

  Table S1 (List of 1,984 human genes up- and downregulated in JHDM1D-AS1-overexpressing AsPC-1 cells)

  XLSX, 5.2M
- Supplemental file 2 -

  Table S2 (List of 1,559 mouse genes up- and downregulated in JHDM1D-AS1-overexpressing AsPC-1 cells)

  XLSX, 222K
- Supplemental file 3 -

  Table S3 (List of 236 probes used in survival analysis using JHDM1D-AS1 signature)

  XLSX, 65K
